# High-Fat Diet-Induced Diabetic Cardiomyopathy in Female Zebrafish: Cardiac Pathology and Functional Decline Mediated by Type 2 Diabetes

**DOI:** 10.3390/nu17132209

**Published:** 2025-07-02

**Authors:** Shuaiwang Huang, Zhanglin Chen, Haoming Li, Yunyi Zou, Bihan Wang, Wenjun Zhao, Lan Zheng, Zuoqiong Zhou, Xiyang Peng, Changfa Tang

**Affiliations:** 1 College of Physical Education, Hunan Normal University, Changsha 410012, China; 202320152962@hunnu.edu.cn (S.H.); zhanglinchen@hunnu.edu.cn (Z.C.); lihaoming@hunnu.edu.cn (H.L.); 202230172024@hunnu.edu.cn (W.Z.); lanzheng@hunnu.edu.cn (L.Z.); 2 Key Laboratory of Physical Fitness and Exercise Rehabilitation of Hunan Province, Hunan Normal University, Changsha 410012, China; 202020151346@hunnu.edu.cn (Y.Z.); xiyangpeng@hunnu.edu.cn (X.P.); 3 Hunan Center for Public Sports Service Research, Hunan Normal University, Changsha 410012, China; wbhxhzz@hunnu.edu.cn

**Keywords:** diabetic cardiomyopathy, high-fat diet, zebrafish model, mitochondrial dysfunction

## Abstract

Background: Diabetic cardiomyopathy (DCM) is characterized by progressive cardiac dysfunction, metabolic dysregulation, myocardial fibrosis, and mitochondrial impairment. Existing animal models, such as streptozotocin (STZ)-induced models, suffer from high mortality and fail to replicate chronic metabolic dysregulation induced by high-fat diets (HFD), whereas HFD or HFD/STZ-combined rodent models require high maintenance costs. This study aimed to establish a zebrafish HFD-DCM model to facilitate mechanistic exploration and drug discovery. Methods: Eighty wild-type female zebrafish were divided into normal diet (N, 6% fat) and HFD (H, 24% fat) groups and fed the diet for 8 weeks. Metabolic phenotypes were evaluated using intraperitoneal glucose tolerance tests and insulin level analysis. Cardiac function was assessed by using echocardiography (ejection fraction, E peak). Structural, metabolic, and oxidative stress alterations were analyzed by histopathology (H&E, Masson, and Oil Red O staining), molecular assays (RT-qPCR, Western blotting), and mitochondrial structure/function evaluations (respiratory chain activity, transmission electron microscopy, and DHE staining). Results: HFD-fed zebrafish developed obesity, insulin resistance, and impaired glucose tolerance. Echocardiography revealed cardiac hypertrophy, reduced ejection fraction, and diastolic dysfunction. Excessive lipid accumulation, upregulated fibrosis/inflammatory markers, impaired mitochondrial respiration, elevated reactive oxygen species levels, and a disrupted redox balance were observed. Conclusions: We established a female zebrafish HFD model that recapitulates human DCM features, including hypertrophy, metabolic dysregulation, fibrosis, inflammation, and mitochondrial dysfunction. This model offers novel insights into DCM pathogenesis and serves as a valuable platform for mechanistic studies and targeted drug screening.

## 1. Introduction

Cardiovascular diseases remain the leading cause of global mortality, with diabetic cardiomyopathy (DCM), an independent complication of type 2 diabetes mellitus (T2DM), drawing significant attention owing to its insidious progression and high mortality rate. Lipotoxicity, a hallmark of cardiac pathology in patients with T2DM, is a critical contributor to cardiomyopathy development [1]. DCM manifests with pathological phenotypes, including cardiac hypertrophy, functional impairment, insulin resistance-driven metabolic dysregulation, inflammatory/fibrotic remodeling, and mitochondrial dysfunction coupled with oxidative stress [2]. The global prevalence of high-fat diets (HFDs) is strongly associated with the increasing incidence of T2DM and DCM [3]. Epidemiological studies have highlighted that chronic HFD consumption elevates diabetes risk by two- to three-fold via obesity, insulin resistance, and chronic low-grade inflammation, while directly exacerbating myocardial lipotoxic injury and heart failure, positioning it as a central driver of early DCM pathogenesis [4].

In T2DM, activation of the sterol regulatory element-binding protein 1c pathway promotes excessive free fatty acid (FFA) uptake in cardiomyocytes. The initial upregulation of fatty acid oxidation and transport genes enhances FFA oxidation, leading to lipid overload and toxic intermediate accumulation (e.g., ceramides and diacylglycerols). Prolonged lipid excess ultimately disrupts mitochondrial β-oxidation and triggers reactive oxygen species (ROS) overproduction [4,5,6]. In T2DM, ectopic lipid deposition impairs insulin signaling by suppressing insulin receptor substrate 1 (IRS-1) phosphorylation and promoting glucose transporter 4 (GLUT4) internalization, thereby forcing the heart to increase reliance on fatty acids for energy production [3,5]. Lipotoxicity further activates the Toll-like receptor 4 (TLR4)/nuclear factor- kappaB (NF-κB) axis, driving macrophage M1 polarization and pro-inflammatory cytokine release (e.g., interleukin-1β [IL-1β], interleukin-18 [IL-18]), while upregulating transforming growth factor- beta1 (TGF-β1) to stimulate fibroblast-to-myofibroblast transition, collectively increasing myocardial stiffness and impairing contractility [3,6,7].

Mounting evidence has identified mitochondrial dysfunction as a pivotal mechanism in DCM pathogenesis [8,9]. Under physiological conditions, cardiomyocytes depend on mitochondrial oxidative phosphorylation for efficient ATP generation to sustain their contractile function [10]. In T2DM, however, excessive FFAs enter cardiomyocytes via cluster of differentiation 36 (CD36) receptors, activating the peroxisome proliferator-activated receptor α (PPARα)/carnitine palmitoyltransferase 1 (CPT1) pathway and overloading mitochondrial β-oxidation. This metabolic strain induces electron transport chain (ETC) electron leakage and ROS bursts [11,12]. ROS accumulation damages mitochondrial DNA (mtDNA) and membrane phospholipids while suppressing sirtuin 3 (SIRT3) deacetylase activity, thereby impairing antioxidant defenses (e.g., superoxide dismutase [SOD] and glutathione peroxidase) and perpetuating oxidative stress [13,14].

Currently, DCM management remains challenging. Streptozotocin (STZ)-induced rodent models of diabetes, traditionally used to study type 1 diabetes, are limited by nephrotoxicity-related mortality and poor recapitulation of T2DM features. Although HFD-or HFD/STZ-combined rodent models better mimic human metabolic dysregulation, they require substantial costs [15,16]. Zebrafish models, with conserved glucolipid regulatory mechanisms and advantages including rapid disease induction, cost-effectiveness, and compatibility with high-throughput screening of lipid metabolism or inflammasome-targeting compounds, emerge as promising tools for DCM research [17,18].

Notably, women with diabetes exhibit higher susceptibility to DCM and heart failure than men with diabetes, with epidemiological evidence indicating that women with diabetes face a five-fold higher risk of heart disease than their non-diabetic counterparts, versus a two-fold increase in diabetic men [19]. This disparity may stem from androgen-mediated cardioprotective effects including antioxidant activity, hypertrophy suppression, and anti-inflammatory responses [20,21,22].

To address the current lack of cost-effective, sex-specific DCM models, this study established a female zebrafish model of diabetic cardiomyopathy induced by HFD. This model was designed to: (1) recapitulate core human DCM pathologies mediated by T2DM and oxidative stress; (2) enable mechanistic investigation of cardiac metabolic dysregulation, mitochondrial dysfunction, and fibro-inflammatory remodeling; and (3) facilitate high-throughput screening of cardioprotective therapeutic agents targeting DCM progression.

## 2. Materials and Methods

### 2.1. Experimental Subjects and Grouping

Healthy 3-month-old female zebrafish (Danio rerio; wild-type AB strain) were obtained from the China Zebrafish Resource Center and maintained under controlled conditions (28 ± 0.5 °C, ≤60% humidity, 14:10 h light/dark cycle). According to the 3R principle for the use of laboratory animals, 80 fish were randomly divided into two groups: normal diet (N, *n* = 40) and high-fat diet (H, *n* = 40). All zebrafish were raised in a breeding system with a density of 5 fish per liter of water. After a week of adaptation, the N group received fresh brine shrimp (6% fat) three times daily (9:00/14:00/18:00), whereas the H group was fed a custom high-fat feed (TP1FM21050, Trophic Animal Feed High-Tech Co., Ltd., Nantong, China; 24% fat) for 8 weeks. The study protocol was approved by the Experimental Animal Ethics Committee of Hunan Normal University (Approval No.: 2019-163).

### 2.2. Intraperitoneal Glucose Tolerance Test (IPGTT) and Biochemical Analysis

After 24 h of fasting, blood was collected from the caudal vein for fasting glucose measurements using a portable glucometer (Yuwell, Danyang, China). The fish were then intraperitoneally injected with glucose (0.5 mg/g body weight), and blood glucose levels were monitored at 30, 90, and 180 min post-injection.

For biochemical analysis, zebrafish were anesthetized with 0.02% MS-222, and dissected with a sterilized scalpel. Skeletal muscle tissue was removed, and homogenates were prepared in saline. Low-density lipoprotein cholesterol (LDL-C) and insulin levels were quantified using commercial kits (Njjcbio, Nanjing, China; MEIAO BIOTECH, Shanghai, China, respectively).

### 2.3. Tissue Collection

After the experimental animals were anesthetized with 0.02% MS-222, hearts were dissected under a stereomicroscope, snap-frozen in liquid nitrogen, and stored at −80 °C for molecular analyses. For histopathology, hearts were fixed in 4% paraformaldehyde (0.1 M, pH 7.4, Servicebio, Wuhan, China) for 12 h. Tissues for Oil Red O and dihydroethidium (DHE) staining were frozen in optimal cutting temperature (OCT) compound (Sakura Finetek, Torrance, CA, USA). Samples for transmission electron microscopy (TEM) were preserved in glutaraldehyde solution (Servicebio, Wuhan, China).

### 2.4. Echocardiography

Cardiac function was evaluated using the Visual Sonics Vevo 2100 echocardiography system (Visual Sonics, Toronto, ON, Canada). Zebrafish were anesthetized with 0.02% MS-222 for 3 min, and ultrasound coupling gel was applied to the cardiac region. A 30 MHz high-frequency probe was used to acquire B-mode and pulsed-wave Doppler recordings, capturing dynamic myocardial contraction and relaxation. Endocardial borders at end-diastole and end-systole were traced from B-mode images to calculate the ejection fraction (EF), fractional shortening (FS), and stroke volume (SV). Linear measurements of the end-diastolic longitudinal diameter (EDD_long_), end-systolic longitudinal diameter (ESD_long_), end-diastolic basal diameter (EDD_base_), and end-systolic basal diameter (ESD_base_) were performed [23]. The E-wave peak velocity (E peak) was derived from pulsed-wave Doppler signals. The operators were blinded to group assignments. Three consecutive measurements were averaged for each fish.

### 2.5. H&E Staining

Cardiac tissues were fixed in 4% paraformaldehyde for 24 h, dehydrated through a graded ethanol series, paraffin-embedded, and sectioned at 5 μm thickness. The sections were mounted on slides (Servicebio, Wuhan, China) and imaged using a NIKON DS-U3 camera control unit (Nikon, Tokyo, Japan).

### 2.6. Masson Staining

Deparaffinized sections were sequentially stained with Weigert’s iron hematoxylin (5 min) and Biebrich scarlet-acid fuchsin (10 min), differentiated in phosphomolybdic acid solution (5 min), and counterstained with aniline blue for collagen fibers (5 min). After dehydration and mounting, the collagen fibers (blue) and cardiomyocytes (red) were visualized under a microscope (Nikon, Tokyo, Japan). The fibrosis area ratio was calculated as (collagen area/total myocardial area) × 100% using ImageJ version win-java8 software.

### 2.7. Oil Red O Staining

Fresh cardiac tissues were embedded in OCT compound (Sakura Finetek, Torrance, CA, USA) and cryosectioned at 10 μm. The sections were pretreated with 60% isopropanol, stained with Oil Red O working solution (15 min), and counterstained with hematoxylin. After mounting with glycerin gelatin, the lipid droplets (red particles) were imaged, and their area percentages were quantified using ImageJ version win-java8 software.

### 2.8. DHE Staining

Fresh cardiac tissues were incubated with DHE staining solution (D7008, Sigma, St. Louis, MO, USA; 1:500 dilution), and nuclei were counterstained with 4′,6-diamidino-2-phenylindole (DAPI). ROS-positive areas fluoresced red and were quantified using ImageJ version win-java8 software.

### 2.9. Transmission Electron Microscopy (TEM)

Cardiac tissues were fixed in 2.5% glutaraldehyde for 6–12 h, rinsed with phosphate-buffered saline (PBS), and post-fixed in 1% osmium tetroxide for 2 h at room temperature (20 ° C). Samples were dehydrated using a graded ethanol series: 30% ethanol (10 min), 50% ethanol (10 min), 70% uranyl acetate in ethanol (3 h or overnight), 80% ethanol (10 min), 95% ethanol (15 min), 100% ethanol (twice, 50 min each), and propylene oxide (30 min). Tissues were infiltrated with propylene oxide/epoxy resin (1:1) for 1–2 h, followed by pure epoxy resin for 2–3 h. After embedding, the samples were polymerized at 40 °C for 12 h and 60 °C for 48 h. Ultrathin sections (70 nm) were mounted on copper grids, stained with uranyl acetate and lead citrate, and imaged using a JEM1400 TEM (JEOL, Tokyo, Japan) equipped with a Morada G3 camera. The sarcomere lengths were measured in six randomly selected fields per sample.

### 2.10. Mitochondrial Respiration Assay

Cardiac tissues were homogenized, and mitochondrial respiratory chain activity was assessed using an Oxygraph-2k high-resolution respirometer (Oroboros, Innsbruck, Austria). After baseline stabilization, substrates and inhibitors were sequentially added: 5 μL of 2 M pyruvate, 10 μL of 400 mM malate, 10 μL of 500 mM ADP, 5 μL of 4 mM cytochrome C, 1 μL of 1 mM carbonyl cyanide m-chlorophenylhydrazone (CCCP), 10 μL of 2 M glutamate, 20 μL of 1 M succinate, 1 μL of 1 mM rotenone, 1 μL of 5 mM antimycin A, 5 μL of 0.8 M ascorbate, and 5 μL of 0.2 M N,N,N′,N′-tetramethyl-*p*-phenylenediamine dihydrochloride (TMPD). Data were collected and normalized to the tissue weight.

### 2.11. Western Blot

Three zebrafish hearts were placed in a single grinding tube to obtain the minimum amount of protein required for Western blot assays. Myocardial tissues were lysed in RIPA buffer (Solarbio, Wuhan, China) containing 1% protease inhibitors. Protein concentrations were determined via BCA assay, and 30 μg of protein per lane was separated by SDS-PAGE and transferred to PVDF membranes. The membranes were blocked with 5% skim milk for 2 h, incubated with primary antibodies overnight at 4 °C, and probed with HRP-conjugated secondary antibodies for 2 h at room temperature. Antibodies included: rabbit anti-β-ACTIN (1:2000), mouse anti-PGC1-α (1:5000), mouse anti-PPARα (1:2000), rabbit anti-CPT1a (1:5000), rabbit anti-GLUT4 (1:4000), rabbit anti-IL-1β (1:2000), rabbit anti-IL-18 (1:4000), rabbit anti-α-SMA (1:2000), rabbit anti-NOX4 (1:8000), rabbit anti- nuclear factor erythroid 2-related factor 2 (NRF2) (1:5000), and rabbit anti-SOD1 (1:5000) (all from Proteintech, Wuhan, China). Bands were visualized using a Tanon imaging system (Tanon, Shanghai, China) and quantified via ImageJ, with β-actin as the loading control.

### 2.12. Real-Time Quantitative PCR

Total RNA was extracted from myocardial tissues using TRIzol (Thermo Fisher Scientific, Waltham, MA, USA) and reverse-transcribed into cDNA (Takara, Tokyo, Japan). qPCR was performed using SYBR Green Premix (Thermo Fisher Scientific, Waltham, MA, USA) and gene-specific primers (Table 1) on a CFX96 system (Bio-Rad Laboratories, Hercules, CA, USA). Cycling conditions: 95 °C for 30 s (initial denaturation); 40 cycles of 95 °C for 5 s and 60 °C for 30 s. Gene expression was normalized to β-actin using the 2^−ΔΔCt^ method.

### 2.13. Statistical Analysis

All data are expressed as mean ± standard error of the mean (SEM). Intergroup differences were analyzed using an unpaired Student’s *t*-test. Graphs were generated using GraphPad Prism 9.0 software. Statistical significance was defined as *p* < 0.05.

## 3. Results

### 3.1. HFD Induces Obesity and Type 2 Diabetes in Zebrafish

**Figure 1:**
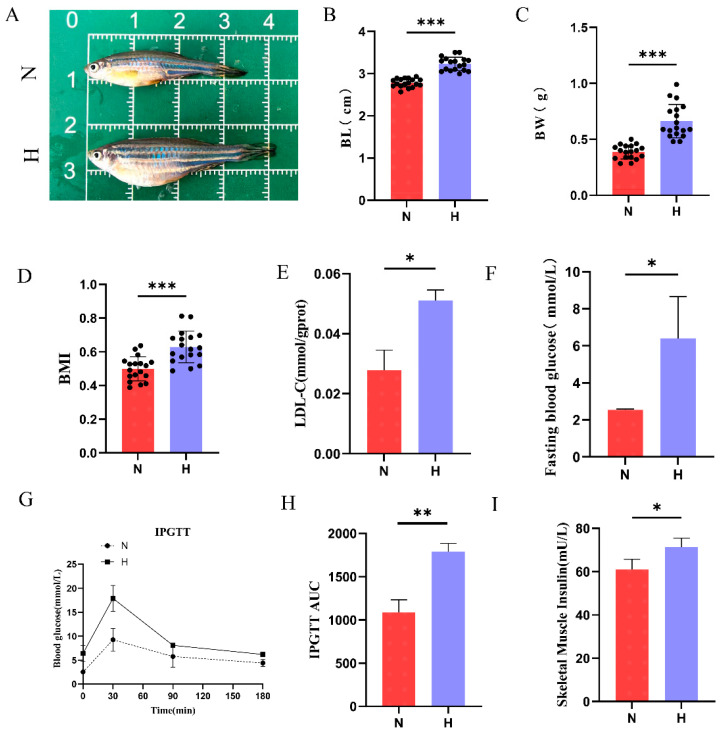
High-fat diet induces obesity and type 2 diabetes in zebrafish. (**A**) Representative images comparing body morphology between the N and H groups. (**B**) Body length of zebrafish after 8-week HFD intervention. (*n* = 18); (**C**) body weight of zebrafish (*n* = 18); (**D**) body mass index of zebrafish (BMI, *n* = 18); (**E**) skeletal muscle low-density lipoprotein cholesterol levels (*n* = 3). (**F**) Fasting blood glucose levels (*n* = 3). (**G**) IPGTT curves (*n* = 3). (**H**) AUC of IPGTT. (**I**) Skeletal muscle insulin content (*n* = 3). * *p* < 0.05, ** *p* < 0.01, *** *p* < 0.001.

Obesity, a primary risk factor for T2DM, was observed in zebrafish after 8 weeks of HFD feeding. Body composition analysis revealed that HFD-fed zebrafish exhibited significantly increased body length, body weight, and body mass index (BMI) compared to the N group (Figure 1A–D). Subsequent biochemical analyses revealed elevated skeletal muscle LDL-C and fasting blood glucose levels in the H group (Figure 1E,F). IPGTT further confirmed impaired glucose metabolism in HFD-fed zebrafish, with persistently higher blood glucose levels throughout the testing period and a significantly larger area under the curve (AUC) than in the N group (Figure 1G,H). Additionally, skeletal muscle insulin levels were markedly elevated in the H group (Figure 1I). Collectively, these findings indicate that an 8-week HFD intervention successfully induced obesity and T2DM phenotypes in zebrafish.

### 3.2. T2DM Induces Cardiac Hypertrophy and Functional Impairment in Zebrafish

**Figure 2:**
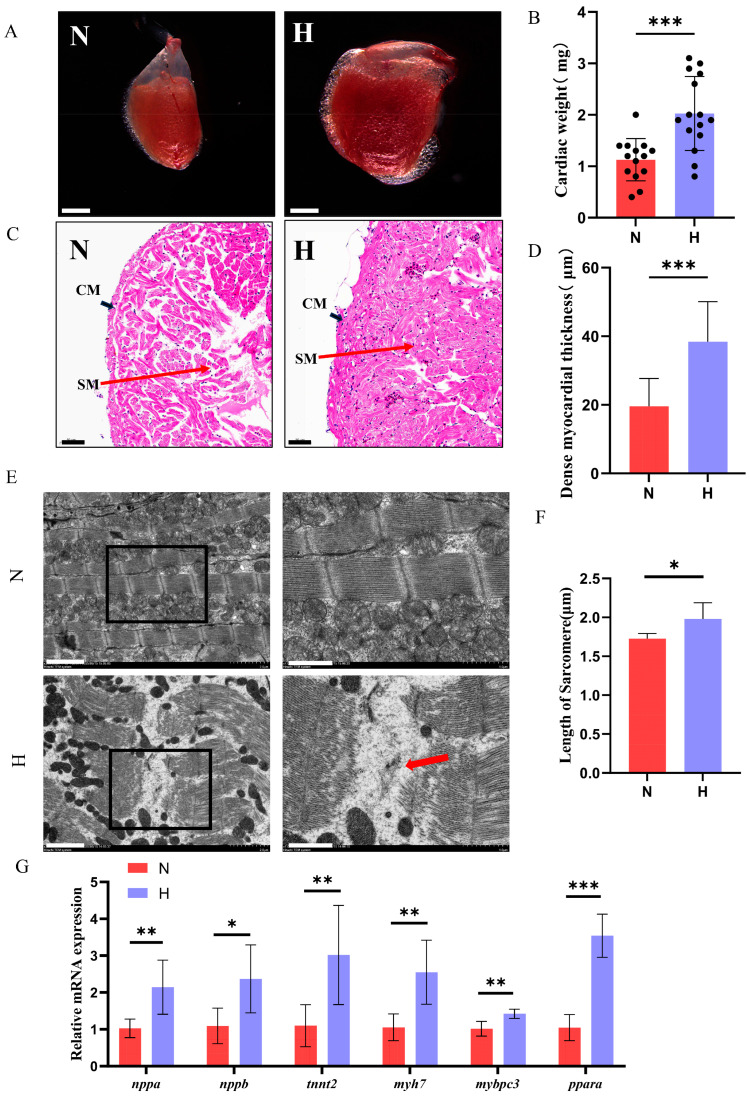
T2DM induces pathological cardiac hypertrophy in zebrafish. (**A**) Representative images of hearts from N and H groups (scale bar: 1 mm). (**B**) Zebrafish heart weight (*n* = 15). (**C**) Hematoxylin and eosin) staining of longitudinal cardiac sections (scale bar: 50 μm). CM, Compact myocardium; SM, Spongy myocardium. (**D**) Compact myocardial thickness (*n* = 3). (**E**) Transmission electron microscopy images of cardiac myofibrils (scale bar: 2 μm; inset shows magnified view of boxed region, scale bar: 1 μm). Red arrows indicate myofibrillar breaks. (**F**) Sarcomere length quantification (*n* = 3). (**G**) Relative mRNA expression levels of *nppa*, *nppb*, *tnnt2*, *myh7*, *mybpc3* and *ppara* in zebrafish hearts (*n* = 6). * *p* < 0.05, ** *p* < 0.01, *** *p* < 0.001.

**Figure 3:**
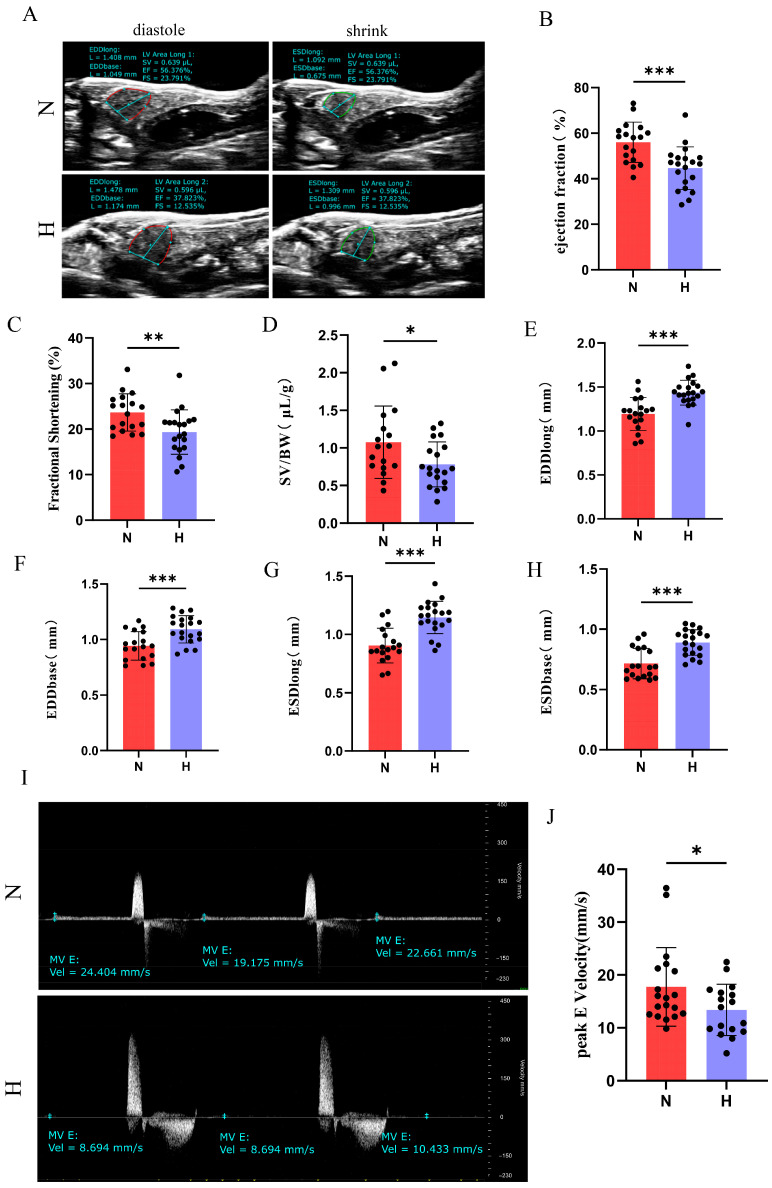
T2DM induces cardiac functional impairment in zebrafish. (**A**) B-mode echocardiography showing end-diastolic and end-systolic cardiac contours. (**B**) Ejection fraction (EF) (*n* = 20). (**C**) Fractional shortening (FS) (*n* = 20). (**D**) Stroke volume normalized to body weight (SV/BW) (*n* = 18). (**E**) End-diastolic longitudinal diameter (EDD_long_) (*n* = 20). (**F**) End-diastolic basal diameter (EDD_base_) (*n* = 20). (**G**) End-systolic longitudinal diameter (ESD_long_) (*n* = 20). (**H**) End-systolic basal diameter (ESD_base_) (*n* = 20). (**I**) Representative pulsed-wave Doppler echocardiographic tracings from groups N and H. (**J**) E-wave peak velocity (E peak) (*n* = 18). * *p* < 0.05, ** *p* < 0.01, *** *p* < 0.001.

To investigate whether T2DM induces pathological cardiac remodeling, stereomicroscopic analysis revealed significantly enlarged hearts in the H group compared to the controls, with increased heart weight (Figure 2A,B). H&E staining demonstrated an expanded maximum longitudinal cross-sectional area and a thickened compact myocardium in HFD-fed zebrafish (Figure 2C,D). Electron microscopy further identified disrupted myofibrillar architecture and elongated sarcomere lengths in HFD hearts (Figure 3E,F). RT-qPCR analysis showed marked upregulation of cardiac stress markers *(nppa, nppb*), contractile dysfunction-related genes (*myh7*, *tnnt2*, *mybpc3*) and a gene related to fatty acids metabolism (*ppara*) in the H group (Figure 2G), consistent with pathological hypertrophy and cardiomyopathy progression [24]. These results indicated that T2DM can lead to pathological changes in zebrafish hearts.

Cardiac functional assessment via echocardiography revealed significant reductions in EF, FS, SV normalized to body weight (SV/BW), and E peak in the H group (Figure 3A–D,I,J). Concurrently, EDD_long_, ESD_long_, EDD_base_, and ESD_base_ significantly increased (Figure 3E–H), indicating ventricular dilation accompanied by impaired systolic and diastolic function. Collectively, these findings demonstrate that 8-week HFD intervention induces pathological cardiac hypertrophy and functional deterioration in zebrafish.

### 3.3. T2DM Disrupts Cardiac Glucolipid Metabolism in Zebrafish

**Figure 4:**
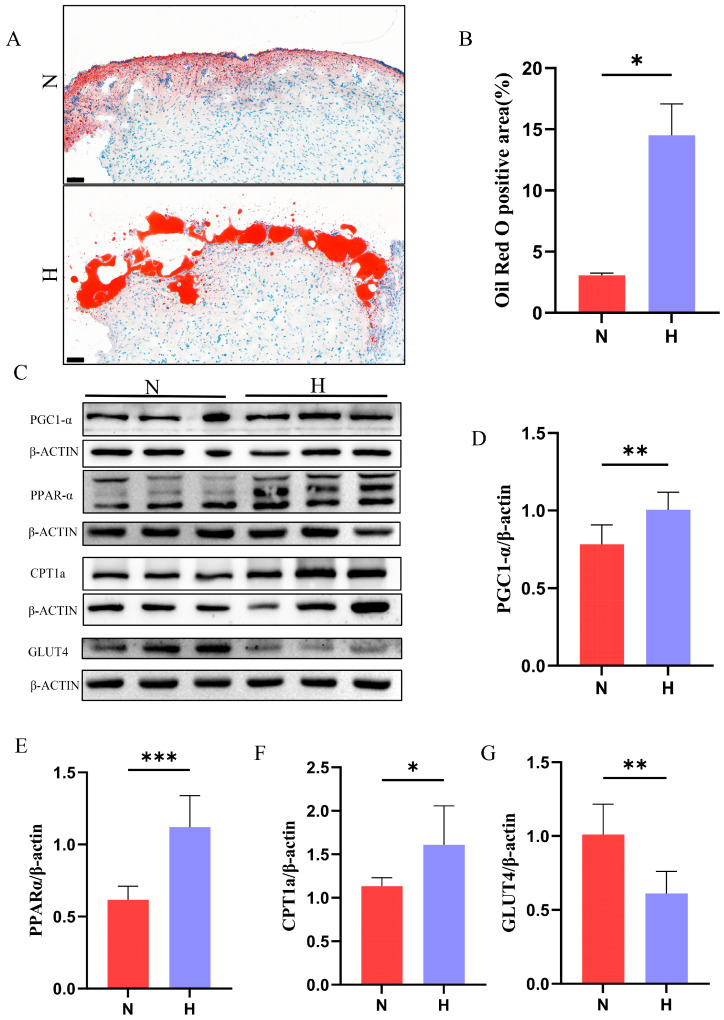
T2DM disrupts lipid and glucose metabolism in zebrafish hearts. (**A**) Oil Red O staining of cardiac tissues after 8-week HFD intervention, with red indicating lipid droplet deposition (scale bar: 50 μm). (**B**) Quantification of Oil Red O-positive areas (*n* = 3). (**C**–**F**) Western blot analysis showing T2DM-induced activation of lipid metabolism-related pathways (PGC1-α/PPARα/CPT1a) and (**G**) downregulation of the glucose metabolism regulator GLUT4 (*n* = 6). * *p* < 0.05, ** *p* < 0.01, *** *p* < 0.001.

Cardiac energy metabolism relies on the balanced utilization of lipids and glucose, with dysregulated substrate preference contributing to metabolic dysfunction. To assess lipid metabolism alterations, Oil Red O staining revealed significantly increased lipid droplet accumulation in the hearts of the H group compared to that in the N group (Figure 4A,B). Western blot analysis demonstrated activation of the PGC1-α/PPARα/CPT1a pathway—a key regulator of fatty acid oxidation [25]—with significantly upregulated expression of these proteins in the H group (Figure 4C–F). Concurrently, marked downregulation of GLUT4 protein expression was observed (Figure 3G). These findings indicate that T2DM induces metabolic reprogramming in zebrafish hearts, characterized by enhanced lipid uptake/oxidation, and suppressed glucose utilization.

### 3.4. T2DM Triggers Cardiac Inflammation and Fibrosis in Zebrafish

**Figure 5:**
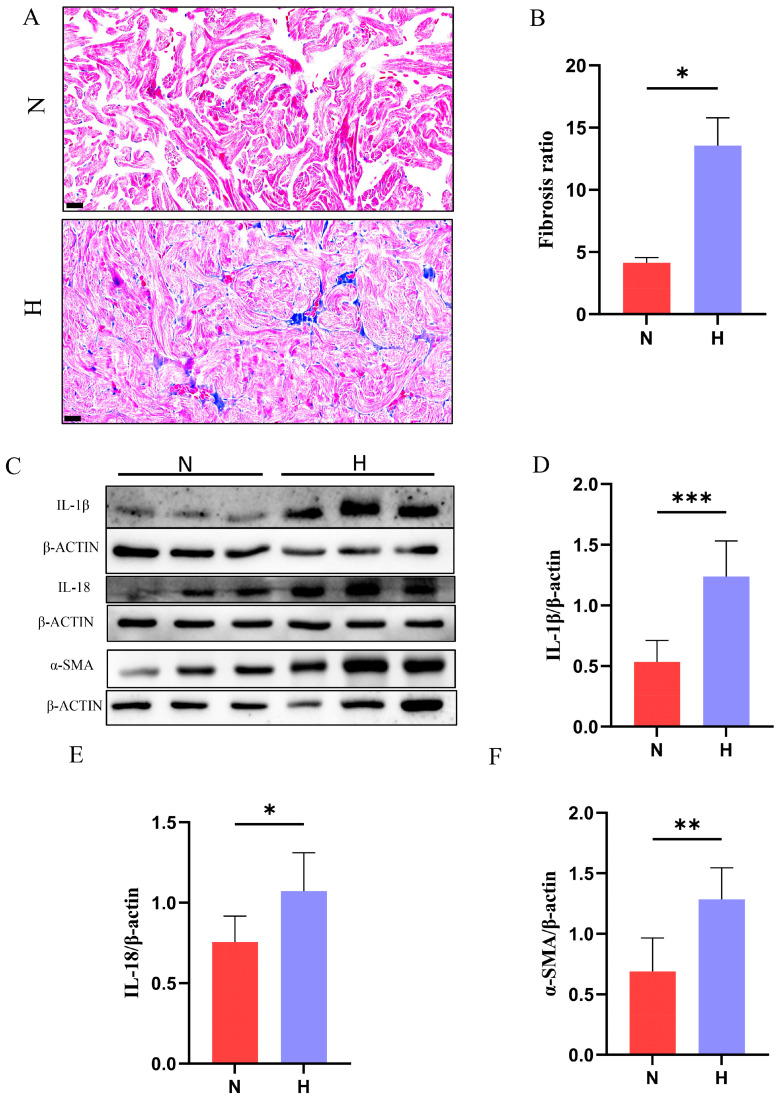
T2DM triggers cardiac inflammation and fibrosis in zebrafish. (**A**) Masson staining of cardiac tissues (blue: collagen fibers; red: cardiomyocytes; scale bar: 20 μm). (**B**) Quantification of the fibrotic area ratio (*n* = 3). (**C**–**F**) Western blot analysis showing T2DM-induced upregulation of pro-inflammatory proteins (IL-1β, IL-18) and the fibrosis marker α-SMA (*n* = 6). * *p* < 0.05, ** *p* < 0.01, *** *p* < 0.001.

To evaluate diabetes-induced cardiac remodeling, Masson staining demonstrated a significant increase in the fibrosis area ratio in HFD hearts compared to that in control hearts (Figure 5A,B). Western blot analysis revealed marked upregulation of the pro-inflammatory cytokines IL-1β and IL-18 (Figure 5C–E), which mediate acute inflammatory responses and adaptive immunity, respectively. Dysregulated activation of these cytokines is closely associated with pathological cardiac injury. Furthermore, α-smooth muscle actin (α-SMA), a hallmark of myofibroblast activation, was significantly elevated in the H group (Figure 5F), directly driving fibrosis progression by enhancing contractility and extracellular matrix (ECM) secretion. Collectively, these results confirmed that T2DM exacerbates inflammatory responses and fibrotic remodeling in zebrafish hearts.

### 3.5. T2DM Impairs Mitochondrial Structure and Function in Zebrafish Hearts

**Figure 6:**
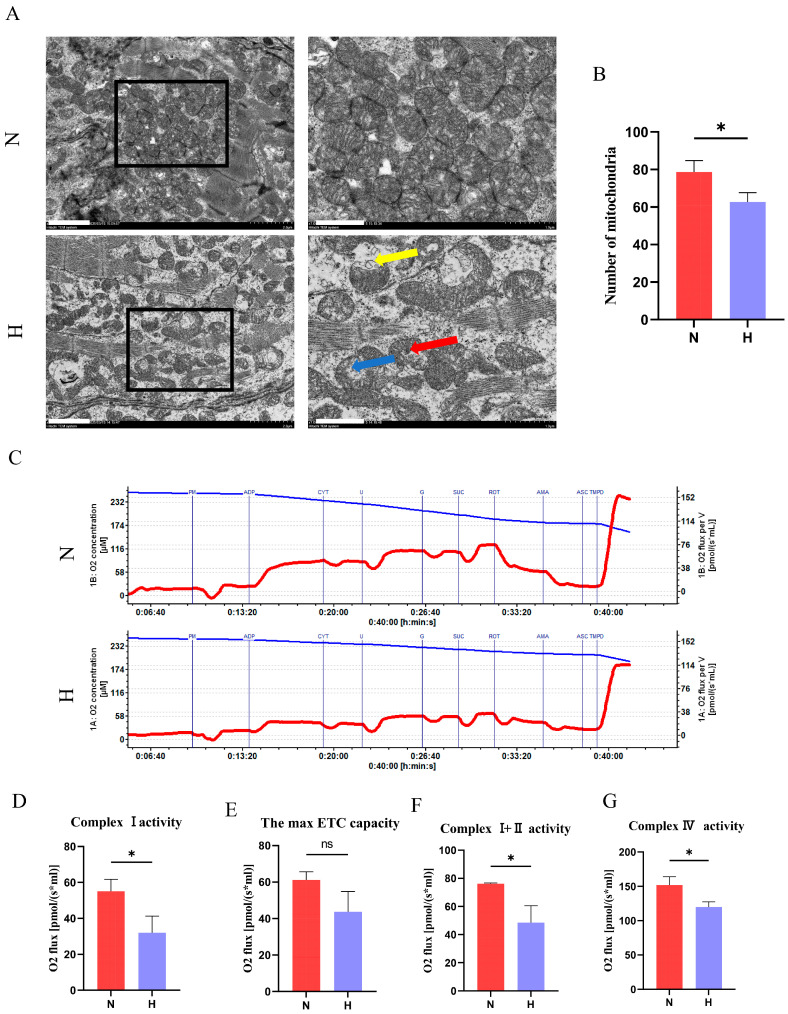
T2DM causes mitochondrial structural damage and dysfunction in zebrafish hearts. (**A**) TEM images of cardiac mitochondria (scale bar: 2 μm; insets show magnified views of boxed regions, scale bar: 1 μm). Red arrows indicate mitochondrial cristae loss, blue arrows denote mitochondrial swelling, and yellow arrows indicate membrane disruption in the H group. (**B**) Quantification of the mitochondrial density per field. (**C**) Mitochondrial respiratory assay traces: the blue curve represents the oxygen concentration, and the red curve reflects the oxygen consumption rate. (**D**) Mitochondrial complex I activity. (**E**) Maximal electron transport chain capacity. (**F**) The combined activity of complexes I and II. (**G**) Mitochondrial complex IV activity. ns, not significant. * *p* < 0.05. (*n* = 3).

Mitochondria are the primary energy-producing organelles in cardiomyocytes and are critically linked to cardiac health. TEM revealed structural abnormalities in the hearts of the H group, including reduced mitochondrial density, cristae loss, swelling, and membrane disruption (Figure 6A). Mitochondrial respiratory chain activity, assessed via high-resolution respirometry, demonstrated diminished complex I, complex I + II, ETCmax, and complex IV activities in the H group (Figure 6C–G), confirming functional impairment.

**Figure 7:**
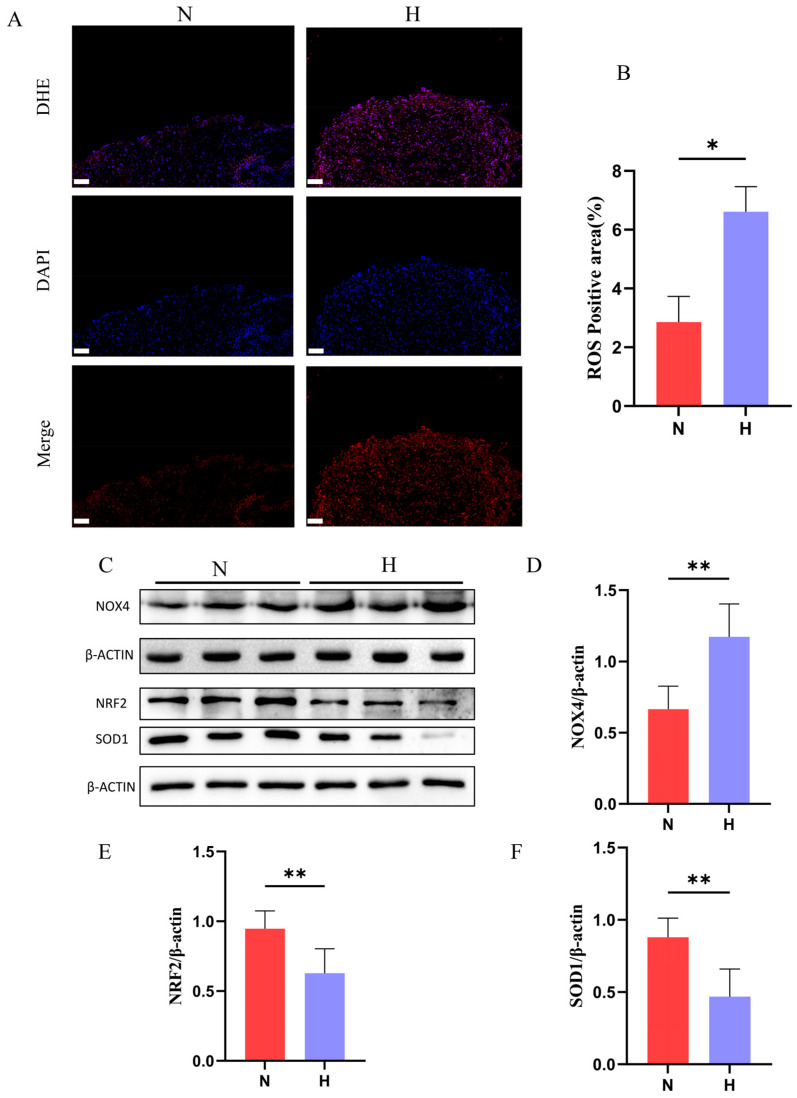
T2DM induces oxidative stress in zebrafish hearts. (**A**) Dihydroethidium staining of the cardiac tissues (red: ROS-positive areas; scale bar: 50 μm). (**B**) Quantification of the ROS accumulation (*n* = 3). (**C**–**F**) Western blot analysis showing T2DM-induced upregulation of the ROS-generating enzyme NOX4 and downregulation of the antioxidant proteins (NRF2, SOD1) (*n* = 6). * *p* < 0.05, ** *p* < 0.01.

As the principal source of ROS, mitochondrial dysfunction exacerbates oxidative stress. Although physiological ROS levels regulate cellular signaling, excessive ROS production drives oxidative damage, senescence, apoptosis, and disease pathogenesis [26]. DHE staining revealed significantly increased ROS-positive areas in the hearts of the H group (Figure 7A,B). Molecular analyses further showed an elevated expression of NADPH oxidase 4 (NOX4), a key ROS-generating enzyme, along with suppressed antioxidant defenses, marked by downregulated NRF2 and SOD1 protein levels in the H group (Figure 7C–F). Collectively, these findings demonstrate that T2DM disrupts mitochondrial architecture and bioenergetics, while inducing a redox imbalance in zebrafish hearts.

## 4. Discussion

DCM, a severe complication of T2DM, involves multifactorial pathogenesis, including cardiac dysfunction, metabolic dysregulation, inflammatory activation, myocardial fibrosis, and mitochondrial structural/functional impairment [2,27]. Glucose tolerance, a key diagnostic marker of T2DM, reflects the body’s capacity to regulate glucose metabolism and detect early-stage metabolic abnormalities [28]. In this study, the 8-week HFD intervention significantly increased the body weight, BMI, fasting blood glucose, and glucose tolerance test AUC in zebrafish, in parallel with elevated skeletal muscle insulin and LDL-C levels. These metabolic alterations closely mirror early-stage T2DM features observed in humans [29]. Lipid metabolism dysregulation emerged as a central mechanism in HFD-induced DCM, with Oil Red O staining revealing excessive myocardial lipid accumulation and upregulated expression of fatty acid oxidation regulators (PPARα, CPT1a). While compensatory activation of lipid oxidation may initially mitigate lipid overload, chronic lipid excess likely promotes toxic intermediate accumulation (e.g., ceramides and diacylglycerols), driving lipotoxicity [1,27]. GLUT4 is an insulin-sensitive glucose transporter predominantly expressed in skeletal muscle, adipose tissue, and cardiomyocytes, and is sequestered in intracellular vesicles under basal conditions. Upon insulin signaling activation or muscle contraction, GLUT4 translocates to the plasma membrane to facilitate glucose uptake, thereby reducing blood glucose levels [30]. Dysregulation of GLUT4 is closely linked to T2DM and insulin resistance, with exercise or pharmacological interventions improving glycemic control by enhancing its activity [31,32,33]. In this study, the H group exhibited upregulated lipid metabolism-related genes and downregulated GLUT4 protein expression in the zebrafish hearts, indicating that T2DM exacerbates cardiac metabolic dysregulation by impairing insulin signaling pathways. This impairment forces the heart to rely on fatty acid uptake and oxidation due to restricted glucose utilization, which further aggravates lipid accumulation.

Echocardiographic results demonstrated a significantly reduced EF and FS in the H group, indicating impaired systolic function. In pulsed-wave Doppler mode, the E-peak, representing early diastolic filling [23,34], markedly decreased, reflecting compromised ventricular diastolic function in T2DM zebrafish. Notably, increased end-diastolic and end-systolic dimensions (EDD, ESD) in the H group hearts, coupled with H&E staining showing expanded cross-sectional areas and thickened compact myocardium, suggested pathological ventricular wall thickening under T2DM conditions. Transmission electron microscopy further revealed elongated sarcomere lengths and myofibrillar disarray in the H group cardiomyocytes, indicative of maladaptive cardiac remodeling. RT-qPCR analysis confirmed the significant upregulation of heart failure markers (*nppa*, *nppb*) and cardiomyopathy-associated genes (*tnnt2*, *myh7*, *mybpc3*). Additionally, increased fibrotic areas in Masson staining and elevated α-SMA expression implied that T2DM likely enhances extracellular matrix deposition, contributing to myocardial stiffness [35,36]. Collectively, these findings provide mechanistic insights into the transition from metabolic dysregulation to structural remodeling during DCM pathogenesis.

Mitochondria, the central organelles involved in cardiac energy metabolism, were severely compromised in the H group zebrafish. Our study revealed reduced mitochondrial density, diminished mitochondrial complex activities, and ultrastructural abnormalities, including cristae loss, swelling, and membrane disruption, in H group hearts, suggesting impaired oxidative phosphorylation. NOX4, a constitutively active ROS-generating enzyme predominantly localized to mitochondria and endoplasmic reticulum in cardiomyocytes, was identified as a major source of H_2_O_2_ overproduction [26,37]. Concurrently, NRF2, a master regulator of antioxidant response elements driving antioxidant gene expression [38]. DHE staining confirmed elevated ROS levels, accompanied by upregulated NOX4 and downregulated NRF2/SOD1 protein expression, indicating T2DM-induced redox imbalance. Mitochondrial dysfunction and ROS overproduction may form a self-perpetuating cycle: lipotoxicity impairs ETC efficiency, reducing ATP synthesis while increasing ROS leakage. Excessive ROS levels further damage mtDNA and membrane integrity, exacerbating the bioenergetic crisis. This mechanism aligns with the mitochondrial pathology observed in clinical DCM patients [27], highlighting mitochondrial protection or antioxidant pathway activation as promising therapeutic strategies for DCM.

This study successfully established a zebrafish DCM model via HFD intervention, recapitulating the core human DCM phenotypes, including compensatory cardiac hypertrophy, functional decline, and lipid deposition. These pathological features are mechanistically linked to inflammatory activation, fibrotic remodeling, and mitochondrial dysfunction, further elucidating the underlying pathophysiology of DCM. This model provides a novel and practical platform to evaluate the cardioprotective efficacy of existing therapies, such as SGLT2 inhibitors. Furthermore, leveraging this zebrafish system for high-throughput screening of small-molecule drugs targeting myocardial energy metabolism may address the current therapeutic limitations in clinical DCM management.

This study provides the first systematic validation of a zebrafish DCM model induced by HFD; however, some limitations remain. The single-ventricular cardiac anatomy of zebrafish differs from that of the human four-chambered heart, which may influence the interpretation of certain functional parameters. Additionally, the relatively short experimental duration (8 weeks) did not fully replicate the chronic progression of human DCM. The exclusive focus on female zebrafish has precluded the exploration of sex-dependent metabolic and cardiac phenotypic variations. Future investigations should prioritize the following directions: (1) extend HFD intervention duration to delineate temporal dynamics of DCM progression and heart failure phenotypes; (2) conduct HFD interventions in both male and female zebrafish to assess sex-specific susceptibility and androgen-mediated cardioprotection; (3) integrate single-cell sequencing to resolve interaction networks among cardiomyocytes, immune cells, and fibroblasts; (4) apply gene-editing tools to dissect molecular mechanisms, therapeutic interventions (e.g., pharmacological or exercise-based), and drug targets within the DCM model.

## 5. Conclusions

This study established an HFD-induced DCM model in female zebrafish that recapitulates critical human DCM pathologies within 8 weeks. HFD triggered systemic metabolic dysfunction (obesity, insulin resistance, and impaired glucose tolerance) and pathological cardiac remodeling, evidenced by hypertrophy, systolic/diastolic dysfunction, lipid accumulation, and suppressed glucose utilization. Mechanistically, mitochondrial damage, ROS overproduction, and redox imbalance were identified as key drivers for the pathology. This model provides a rapid, pathologically relevant platform for the study of the DCM mechanism and screening for cardioprotective drugs.

## Figures and Tables

**Table 1:** Primer Sequence.

Gene	Forward	Reverse
*Glyceraldehyde-3-phosphate dehydrogenase (gapdh)*	ATCATCTCTGCCCCAAGTGC	ACGGTCTTCTGTGTTGCTGT
*Natriuretic peptide A (nppa)*	ACAGAGACCGAGAGGAAG	AGGGTGCTGGAAGACCCTAT
*Natriuretic peptide B (nppb)*	CATGGGTGTTTTAAAGTTTCTCC	CTTCAATATTTGCCGCCTTTAC
*Peroxisome proliferator-activated receptor alpha (ppara)*	ATGTCCCACAATGCCATCCG	TCTGCTTGGCCAGGGTTTTC
*Troponin T2, cardiac type (tnnt2)*	GAAGGCCAGTGAAATGTGGC	CGACCTTTGGCACTCTGGT
*Myosin heavy chain 7 (myh7)*	CTTGGTGCACATCAGACAAGG	CTGGGGGTGAATGTCAGCTT
*Myosin-binding protein C, cardiac-type (mybpc3)*	CTCCACCAGCGAGCCTATTG	ACGTCTCTCATTTCTTGATGTCT

## Data Availability

The original contributions presented in this study are included in the article. Further inquiries can be directed to the corresponding authors.

## Abbreviations

DCM	Diabetic Cardiomyopathy
HFD	High-Fat Diet
T2DM	Type 2 Diabetes Mellitus
FFA	Free Fatty Acid
STZ	Streptozotocin
BMI	Body Mass Index
RT-qPCR	Real-Time Quantitative Polymerase Chain Reaction
mtDNA	Mitochondrial DNA
ROS	Reactive Oxygen Species
ECM	Extracellular Matrix
EF	Ejection Fraction
FS	Fractional Shortening
EDD	End-Diastolic Diameter
ESD	End-Systolic Diameter
SV	Stroke Volume
IPGTT	Intraperitoneal Glucose Tolerance Test
AUC	Area Under the Curve
LDL-C	Low-Density Lipoprotein Cholesterol
GLUT4	Glucose Transporter Type 4
ETC	Electron Transport Chain
IL-1β	Interleukin-1 Beta
IL-18	Interleukin-18
α-SMA	Alpha-Smooth Muscle Actin
IRS-1	Insulin Receptor Substrate 1
NF-κB	Nuclear Factor Kappa-B
NOX4	NADPH Oxidase 4
NRF2	Nuclear Factor Erythroid 2-Related Factor 2
PGC1-α	Peroxisome Proliferator-Activated Receptor Gamma Coactivator 1-alpha
PPARα	Peroxisome Proliferator-Activated Receptor Alpha
CPT1a	Carnitine Palmitoyltransferase 1A
SIRT3	Sirtuin 3
SOD	Superoxide Dismutase
TGF-β1	Transforming Growth Factor-Beta1
TLR4	Toll-Like Receptor 4
